# Levels of Daily Light Doses Under Changed Day-Night Cycles Regulate Temporal Segregation of Photosynthesis and N_2_ Fixation in the Cyanobacterium *Trichodesmium erythraeum* IMS101

**DOI:** 10.1371/journal.pone.0135401

**Published:** 2015-08-10

**Authors:** Xiaoni Cai, Kunshan Gao

**Affiliations:** State Key Laboratory of Marine Environmental Science, Xiamen University, Xiamen, Fujian, China; CEA-Saclay, FRANCE

## Abstract

While the diazotrophic cyanobacterium *Trichodesmium* is known to display inverse diurnal performances of photosynthesis and N_2_ fixation, such a phenomenon has not been well documented under different day-night (L-D) cycles and different levels of light dose exposed to the cells. Here, we show differences in growth, N_2_ fixation and photosynthetic carbon fixation as well as photochemical performances of *Trichodesmium* IMS101 grown under 12L:12D, 8L:16D and 16L:8D L-D cycles at 70 μmol photons m^-2^ s^-1^ PAR (LL) and 350 μmol photons m^-2^ s^-1^ PAR (HL). The specific growth rate was the highest under LL and the lowest under HL under 16L:8D, and it increased under LL and decreased under HL with increased levels of daytime light doses exposed under the different light regimes, respectively. N_2_ fixation and photosynthetic carbon fixation were affected differentially by changes in the day-night regimes, with the former increasing directly under LL with increased daytime light doses and decreased under HL over growth-saturating light levels. Temporal segregation of N_2_ fixation from photosynthetic carbon fixation was evidenced under all day-night regimes, showing a time lag between the peak in N_2_ fixation and dip in carbon fixation. Elongation of light period led to higher N_2_ fixation rate under LL than under HL, while shortening the light exposure to 8 h delayed the N_2_ fixation peaking time (at the end of light period) and extended it to night period. Photosynthetic carbon fixation rates and transfer of light photons were always higher under HL than LL, regardless of the day-night cycles. Conclusively, diel performance of N_2_ fixation possesses functional plasticity, which was regulated by levels of light energy supplies either via changing light levels or length of light exposure.

## Introduction

*Trichodesmium*. spp are the most abundant dizaotrophic cyanobacteria [1] in oligotrophic tropical and subtropical oceans [2]. They provide biologically available nitrogen sources and often form large extensive blooms. Current estimates of marine nitrogen fixation ranging from 100 to 200 Tg (1–2×10^14^ g) yr^-1^, with *Trichodesmium* contributing about half of the total [3].

Diazotrophs have developed specific molecular and physiological strategies to protect nitrogenase from O_2_ evolved during photosynthesis [4–9] since nitrogenase is extremely sensitive to O_2_. While some unicellular diazotrophic cyanobacteria fix N_2_ at night to avoid photosynthetic oxygen inhibition of the nitrogenase complex (temporal separation), many filamentous diazotrophic cyanobacteria develop specialized N_2_-fixing cells, heterocysts, with thickened cell walls, which do not evolve O_2_ [10, 11]. However, the non-heterocystous *Trichodesmium*. spp allow photosynthesis and N_2_ fixation to proceed simultaneously during the daytime in the same trichome, and the mechanisms involved are intriguing and controversial [12]. N_2_ fixation and photosynthesis in *Trichodesmium* may be controlled by a circadian rhythm since it possesses the “clock” genes (kaiABC) [13, 14]. *Trichodesmium thiebautii* shows circadian patterns of N_2_ fixation in parallel with the transcription of *nifH* [15]. The changes in nitrogenase activity in *Trichodesmium* can reflect light-dependent activation and deactivation of the Fe protein [16]. Moreover, respiration, photosynthetic O_2_ evolution and nitrogen fixation all show some correlated diurnal variations with a peak in nitrogen fixation at midday corresponding to a dip in photosynthetic O_2_ evolution [17]. However, little is known about the regulation of photosynthetic carbon fixation and nitrogen fixation activity upon exposing *Trichodesmium* to different light-dark regimes.

Since N_2_ fixation requires energy derived via photosynthesis but evolved O_2_ inhibits it in *Trichodesmium*, we hypothesize that both light intensity and length of the light period (light dose) would affect its diurnal patterns and levels of N_2_ fixation. We therefore investigated how N_2_ fixation and photosynthetic carbon fixation in the marine non-heterocystous cyanobacterium *Trichodesmium erythraeum* IMS101 respond to changes in light-dark cycles under different light levels. We report here that the activity of N_2_ fixation oscillated diurnally with differential peaking time under different light regimes, which was the latest for the shortest light period and contrary to that of photosynthetic carbon fixation.

## Materials and Methods

### Culture condition and experimental set-up

*Trichodesmium erythraeum* IMS101, originally isolated from the North Atlantic Ocean, were grown in 1 L glass flasks (500–800 ml cultures) in YBC-II medium without combined nitrogen source [18]. The culture was maintained in a plant growth chamber (GXZ, Ningbo, China) at 25°C under 70 ± 5 (LL) and 350 ± 19 (HL) μmol photons m^-2^ s^-1^ PAR (photosynthetically active radiation, 400–700 nm), representing sub-saturating and super-saturating values for *Trichodesmium* according to [19]. The light was supplied by white fluorescent tubes (Philips) within the chamber. These two different light levels were achieved by using neutral density screen, and were measured using a LI-COR 2π PAR sensor (PMA2100, Solar light, USA). Three independent cultures under each light level and each light regime were run and used to measure the growth rate and all subsequent physiological parameters.

The cultures were initially run with a 12L:12D (Light: Dark) cycle under the two light levels for 60 generations (more than 180 days) before being shifted to 8L:16D or 16L:8D regime. Under each light regime, subsequently, the cultures were run in triplicates and semi-continuously diluted every 4–5 days to achieve steady-state exponential growth for 50–55 days (10–40 generations) prior to the measurements of growth, N_2_ fixation and photosynthetic parameters. The measurements of N_2_ and carbon fixation were carried out in 2 h intervals during the light period and at the onset of the dark period.

Biomass of the cultures was estimated by measuring chlorophyll *a* concentration. Even though the chl *a* content per cell would be different under different regimes or different light levels, the content was shown to be constant after the cells had been acclimated to different light levels in semi-continuously diluted cultures of the same strain [19]. Therefore, the growth rate under each light regime was estimated as μ = (lnC_2_-lnC_1_)/(t_2_-t_1_), where C_1_ and C_2_ represent the chl *a* concentrations per ml at time t_1_ (after dilution) and t_2_ (before the next dilution), respectively. Chl *a* contents were determined by filtering samples onto glass-fiber filters (GF/F, Whatman) and then extracted in 4 ml 100% methanol at 4°C overnight and quantified from the absorption spectra (400–700 nm) of the supernatants with a spectrophotometer (DU800, Beckman, USA). The chl *a* concentration in the supernatant was estimated according to the equation: [Chl *a*] (μg mL^−1^) = 12.94 × (A_665_ − A_750_) [20].

### N_2_ fixation

Rate of N_2_ fixation were measured in sequence with 2 h intervals using the acetylene reduction assay (ARA) [21]. Samples of 5 ml subculture were placed in 13 ml glass vials. Gas-tight syringes were used to inject 1ml acetylene into the headspace. The vials were incubated for 1 h under the growth conditions with continual shaking, a 500 μl headspace sample was then analyzed in a gas chromatograph equipped with a flame-ionization detector and quantified relative to an ethylene standard. The ethylene produced was calculated using the Bunsen gas solubility coefficients according to [22], and the ethylene production to N_2_ fixation conversion factor of 4 was used to derive N_2_ fixation rates, which were normalized to chl *a*.

### Carbon fixation

Subsamples of 20 ml were taken every 2 h to measure the carbon fixation rate in parallel with the N_2_ fixation measurements. The incubation (1 h) was initiated by inoculating 100 μL (5 μCi) of NaH^14^CO_3_ (ICN Radiochemicals, Irvine, California, USA) and maintained under the growth conditions. The cells were collected onto Whatman GF/F glass fiber filters (Φ 25 mm) and stored at -20°C until analysis. To determine the radioactivity, the filters were exposed to HCl fumes overnight and dried at 60°C to get rid of non-assimilated C^14^ before being digested in scintillation cocktail (Hisafe 3, Perkin-Elmer, Shelton, CT, USA), and measured with a scintillation counter (Tri-Carb 2800TR, Perkin-Elmer, Shelton, CT, USA), as previously descripted [23].

### Fluorescence

Fluorescence parameters was measured every 2 h using a Fluorescence Induction and Relaxation Fluorometer (FIRe, Satlantic, Halifax, Canada) with a green (530 nm with 30 nm bandwidth) excitation light. A saturating (5×10^4^ μmol photons m^-2^ s^-1^), single turnover flash (120 μs) was applied and the actinic light was set at the growth light level. The quantum yield of PSII was calculated as ΦPSII = (F_M_'−F_S_)/F_M_', where the F_S_ and F_M_' were the steady state and maximum chlorophyll fluorescence measured under the light, respectively. In the dark period, the quantum yield was measured without actinic light. The quantum yield was estimated as F_V_/F_M_ = (F_M_−F_O_)/F_M_, where the F_O_ and F_M_ were the minimal and maximum chlorophyll fluorescence measured in the darkness. The relative ETR (rETR) was calculated as rETR = ΦPSII× Growth PAR.

### Statistics

Triplicate cultures were operated under each light level and/or each day-night cycles, and all of the data were obtained with 3 replication from the triplicate cultures. The data were expressed as mean value ± SD for three independent cultures. The daily fixed amount of carbon was derived by integrating the carbon fixation rate during the light period under the different L-D regimes and light levels, while the daily fixed amount of N_2_ was derived by integrating the N_2_ fixation rate during the light period and at the onset of the dark period. Daily light dose was calculated as I_eq_×daytime length (s). One-way ANOVA and Tukey test were used to establish differences among the treatments at a significance level of p< 0.05.

## Results

### Growth

**Fig 1:**
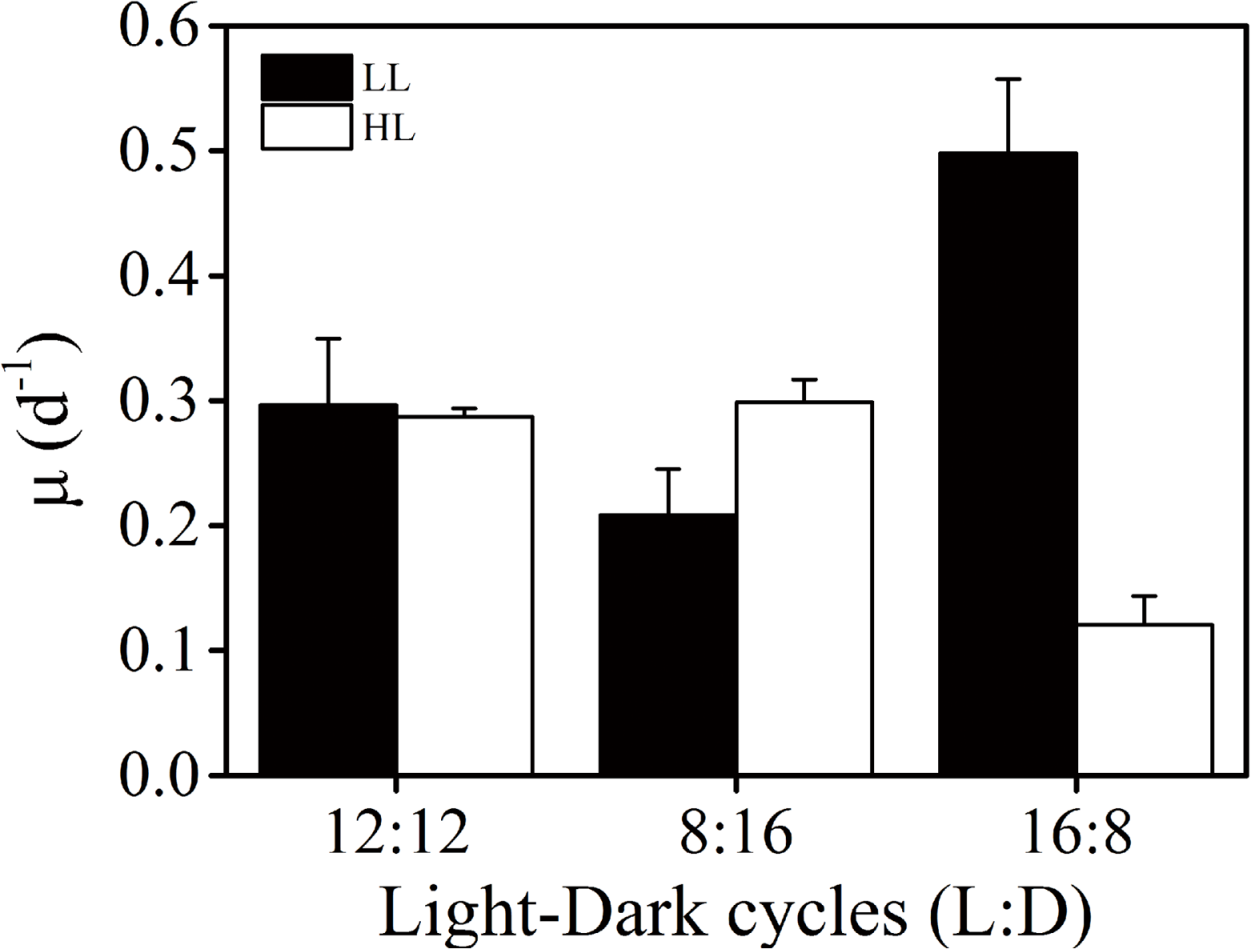
Specific growth rate of *Trichodesmium* IMS101. Growth rates of *Trichodesmium* IMS101 of LL (70 μmol photons m^-2^ s^-1^, dark column) and HL (350 μmol photons m^-2^ s^-1^, open column) cultures grown under different Light Dark (LD) regimes. Values represent mean ± SD of triplicate cultures under each regime.

Under the light regime of 12L:12D, growth rate of *Trichodesmium* IMS101 did not show significant difference between 70 (LL) and 350 (HL) μmol photons m^-2^ s^-1^ (p = 0.83) (Fig 1). After the LL and HL-grown cells had been transferred to 8L:16D regime for 50 days, the growth rate of LL-grown cells was significantly reduced by 30% (P = 0.02) compared to that of 12L:12D regime, while that of the HL was not affected (P = 0.94). When the light period was extended to 16 h (dark period to 8 h), the growth rate significantly increased in the LL-grown cells by 68% (P = 0.006), but was significantly reduced by 57% in the HL-grown ones (P = 0.001), compared to that in the 12L:12D regime (Fig 1).

## N_2_ fixation

**Fig 2:**
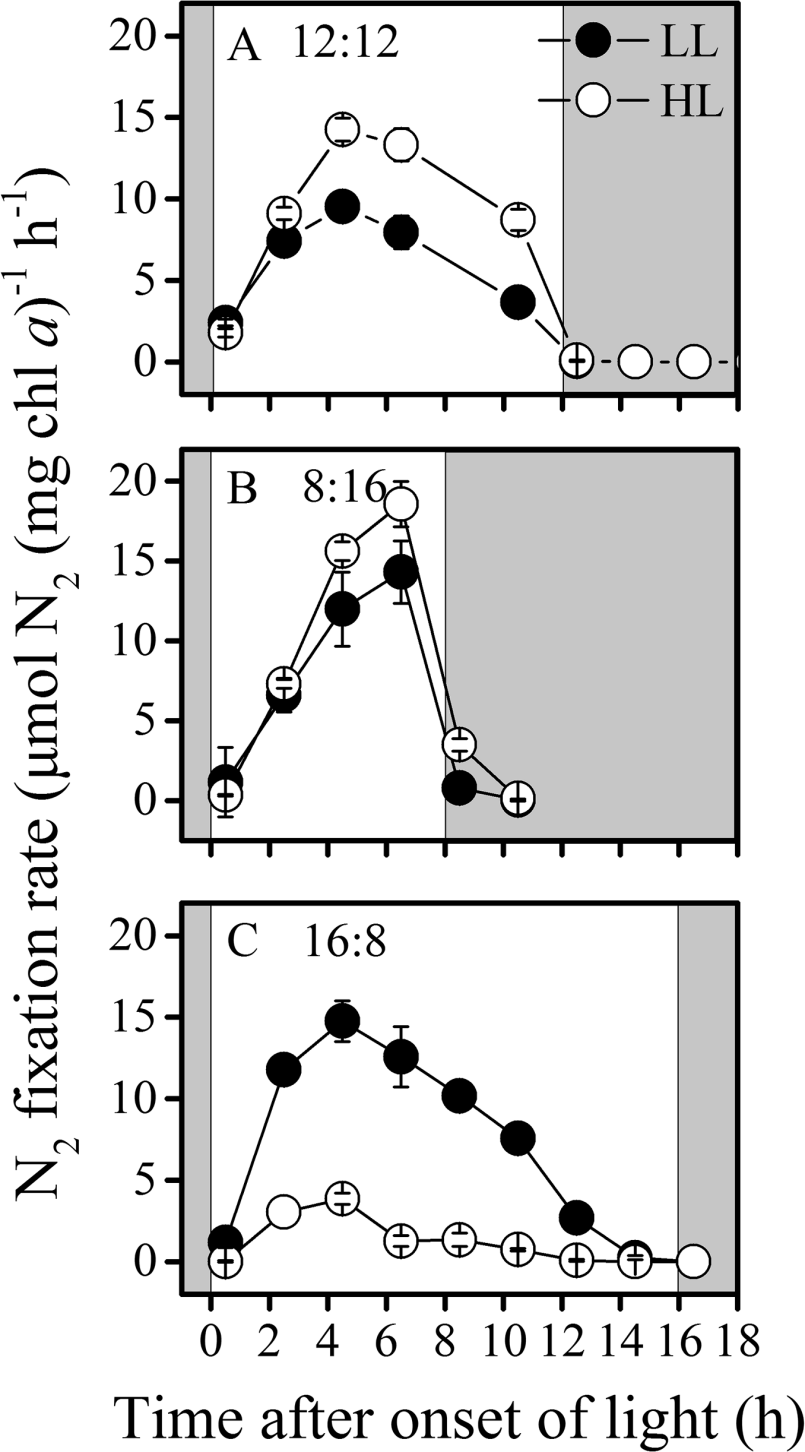
Diel variations of N_2_ fixation. Diel variations of N_2_ fixation of LL (closed circles) and HL (open circles) cultures acclimated to different LD regimes. (A) 12L:12D, (B) 8L:16D and (C) 16L:8D regimes. Shaded areas represent dark periods. Values represent mean ± SD of triplicate cultures under each regime.

After the cells had been grown under the regimes of 8L:16D and 16L:8D, respectively, for 50 days, the N_2_ fixation rates showed distinct variations during the light period under both LL and HL conditions (Fig 2). The LL- and HL- grown cells showed similar diurnal patterns of N_2_ fixation (Fig 2). Under the 12L:12D regime, cells began to fix N_2_ shortly after the onset of the light with the rate peaked around 4.5 h into the light period, and then decreased gradually to zero at the beginning of the dark period (Fig 2A). Shortening the light period to 8 h, the peak of N_2_ fixation was reached later during the day (Fig 2B), with the N_2_ fixation peaked at the end of light period and extended to dark period (1 h after darkness onset). In addition, the maximal N_2_ fixation rates were substantially higher by 50% (p = 0.006) under LL and by 28% (p = 0.08) under HL compared to that of 12L:12D cultures, respectively (Table 1). Nevertheless, the total daily N_2_ fixation was lower than that in 12L:12D cultures. In contrast, when the light period was extended to 16 h (16L:8D), the N_2_ fixation peaked at the same time (4.5 h into the light period) as that in 12L:12D (Fig 2C). However, elongation of the light period led to 3.8 times higher maximal N_2_ fixation rates in the LL grown cells than that in the HL grown ones (Fig 2C), the amount of N_2_ fixed in 24 h of LL grown cells were 5 times higher than HL cells (Table 1).

**Table 1:** Means or daily N_2_ and carbon fixation and ratios of C/N.

LD regime	Light level	Maximum N_2_ fixation rate	Total N_2_ fixed in the light	Total N_2_ fixed in 24 hours	Percentage of N_2_ fixed in the dark	Maximum C fixation rate	Total C fixed in the light	C:N_2_ fixation ratio in the light
(h:h)		(μmol N_2_ (mg chl *a*)^-1^ h^-1^)	(μmol N_2_ (mg chl *a*)^-1^)	(μmol N_2_ (mg chl *a*)^-1^)	(%)	(μmol C (mg chl a)^-1^ h^-1^)	(μmol C (mg chl a)^-1^)	(mol:mol)
**12:12**	LL	9.5±0.8	71.0±9.0	71.0± 9.0	0	282.3±51.9	2457.3±22	34.6
	HL	14.3±0.7	114.8±4.2	114.8± 4.2	0	397.4±9.1	3274.3±93	28.5
**8:16**	LL	14.3±2.0	66.5±12.9	68.6± 16.0	2.99	110.6±5.0	616.4±22	9.3
	HL	18.6±1.4	84.2± 11.7	90.5± 9.4	6.96	302.0±84.5	1658.5±310	19.7
**16:8**	LL	14.8±1.3	120.8± 10.0	120.8± 10.0	0	273.6±34.1	2574.4±316	21.3
	HL	3.9±0.4	20.7 ±1.9	20.7± 1.9	0	594.2±44.1	4966.8±290	239.9

Means of daytime or daily N_2_ fixation and carbon fixation (± SD) under different light: dark (LD) regimes and ratios of C/N assimilation during daytime. The C:N_2_ fixation ratio were based on the amount of carbon and N_2_ fixed in the light period.

### Carbon fixation

**Fig 3:**
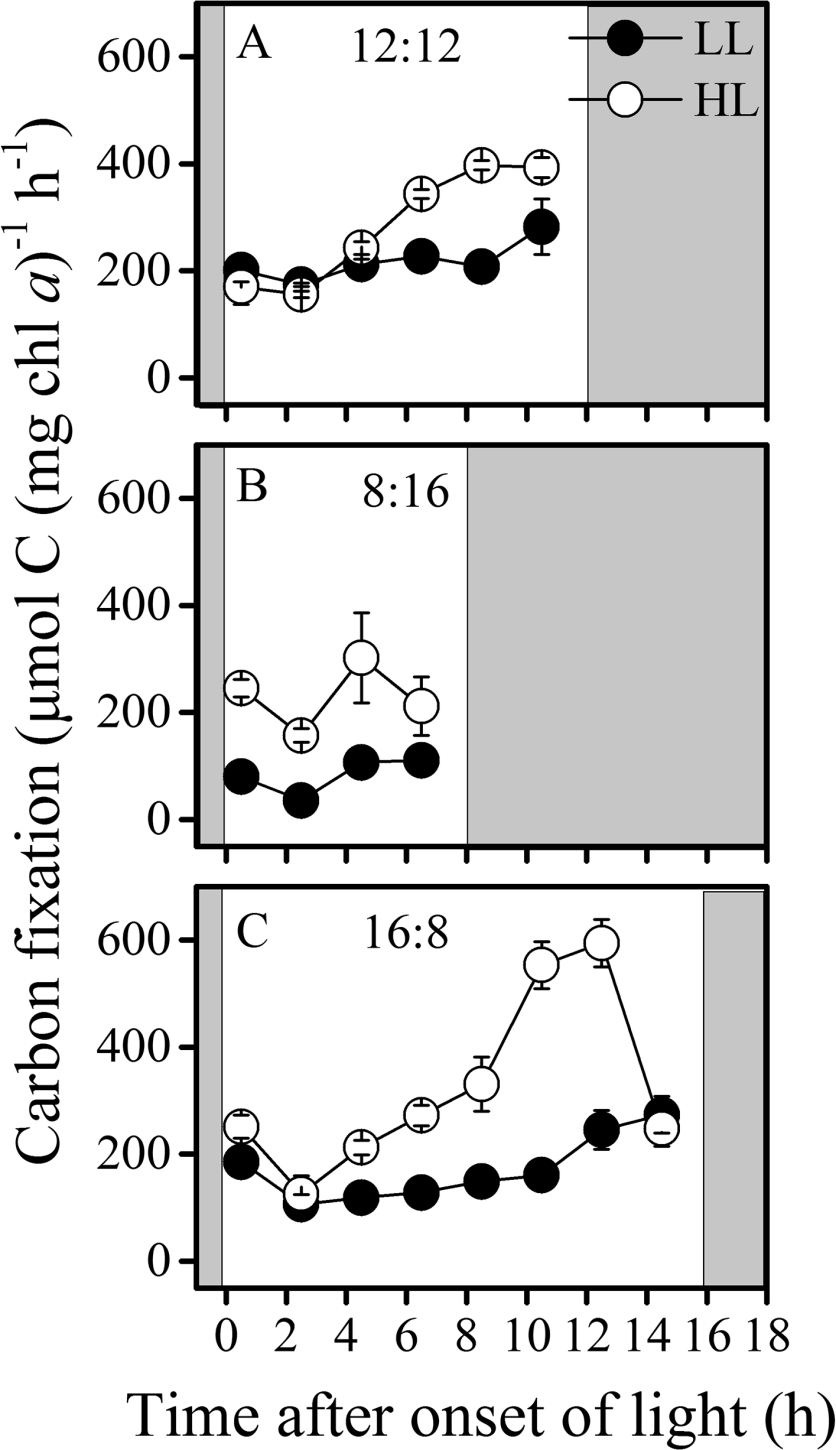
Diel variations of carbon fixation. Diel variations of carbon fixation of LL (closed circles) and HL (open circles) cultures acclimated to different LD regimes. (A) 12L:12D, (B) 8L:16D and (C) 16L:8D regimes. Shaded areas represent dark periods. Values represent mean ± SD of triplicate cultures under each regime.

The carbon fixation rates were higher in HL-grown cells compared to LL-grown cells in all light regimes (Fig 3). In the HL-grown cells, carbon fixation decreased first, dipped around 2.5 h into the light period and then increased, peaked at the 2–4 h before the light was off. However, in the LL grown cells, the carbon fixation rate first decreased at the onset of light period and then gradually increased toward the end of light period regardless of the light regimes (Fig 3). In terms of the total amount of carbon fixed in light, extending the light period to 16 h significantly increased the amount of carbon fixed by 50% (p< 0.001) in HL-grown cells compare to that under 12L:12D regime, and increased by 4% (p = 0.26) in LL-grown cells compared to that under 12L:12D regime. The amplitude and periodicity of carbon fixation of LL-exposed cultures varied much less than those of HL cultures. The rates and amounts of total carbon fixed in light as well as C:N assimilation ratio were the lowest in LL-grown cells at 8L:16D regime, while the highest values were found in the HL-grown cells at 16L:8D regime (Table 1).

### Fluorescence

**Fig 4:**
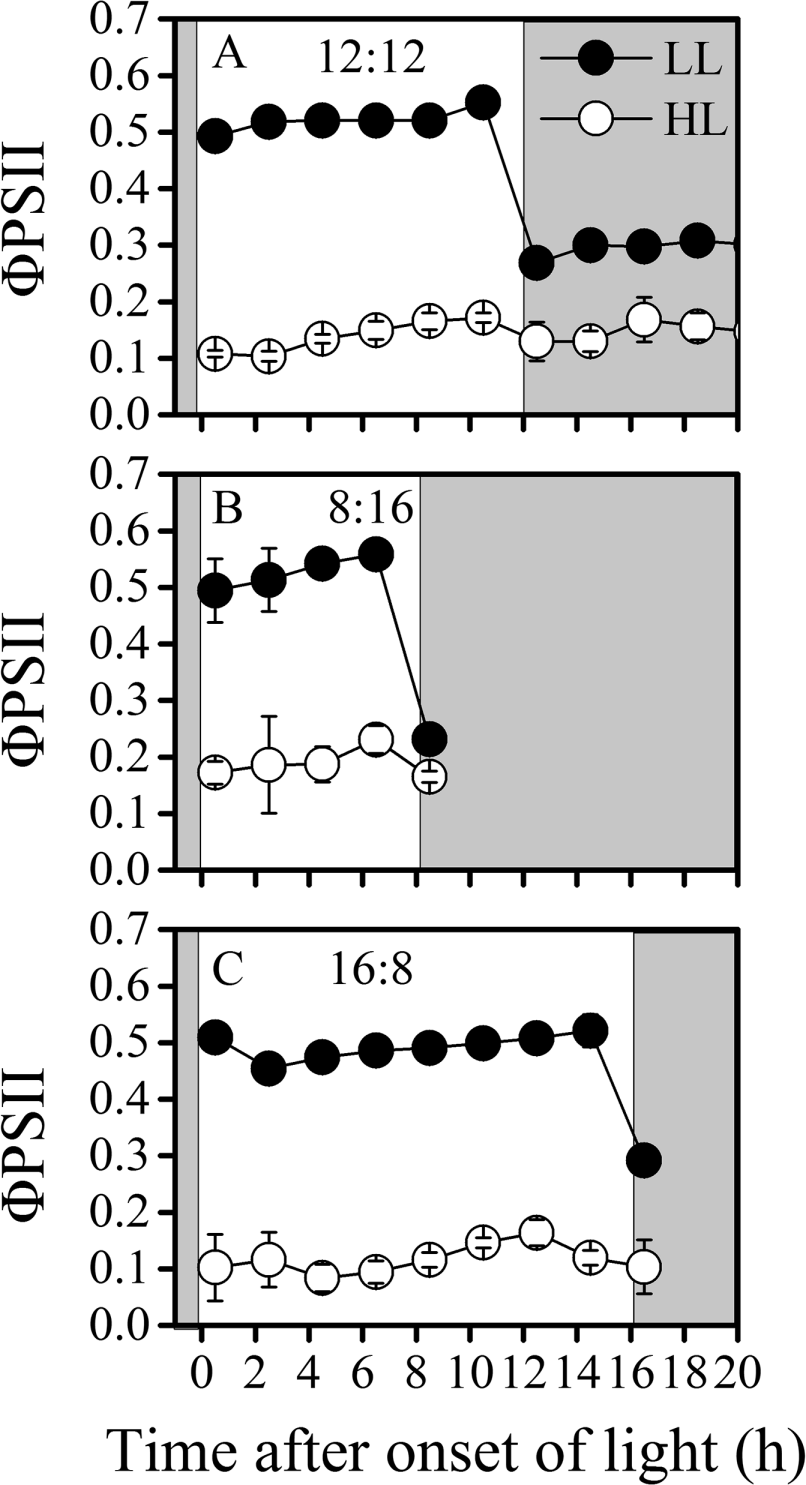
Diel variations of ΦPSII. Diel variations of quantum yield of PSII (ΦPSII) of low light (LL, closed circles) and high light (HL, open circles) cultures acclimated to different LD regimes, (A) 12L:12D, (B) 8L:16D and (C) 16L:8D. Shaded areas represent dark periods. Values represent mean ± SD of triplicate cultures under each regime.

**Fig 5:**
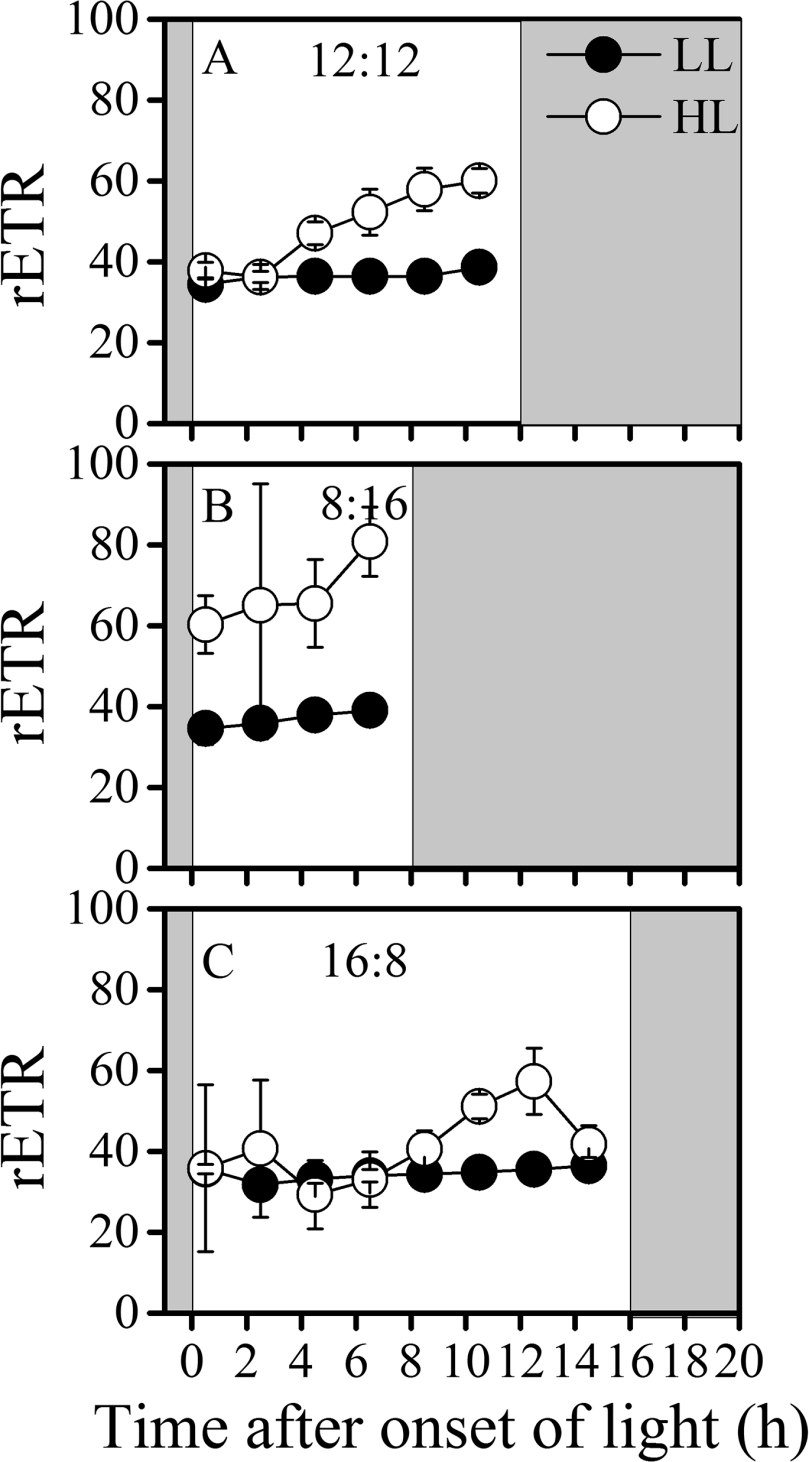
Diel variations of rETR. Diel variations of relative ETR of low light (LL, closed circles) and high light (HL, open circles) cultures acclimated to different LD regimes, (A) 12L:12D, (B) 8L:16D and (C) 16L:8D. The values of rETR was determined from the instant ΦPSII multiplied by growth light irradiances. Shaded areas represent dark periods. Values represent mean ± SD of triplicate cultures under each regime.

The effective quantum yield (Φ_PSII_) (Fig 4) and relative electron transport rate (rETR) (Fig 5) showed similar patterns among the three light-dark regimes. The effective quantum yield (Φ_PSII_) of LL cells was about 5 times higher than HL cells (Fig 4), while the maximal rETR were about 1.5 to 2 times higher in HL than in LL-grown cells. In terms of the diel variations, in 12L:12D and 8L:16D regimes, Φ_PSII_ of both LL and HL-grown cells gradually increased toward the end of light period, once in the dark period, the quantum yield dramatically decreased. In the 16L:8D cultures, Φ_PSII_ of LL-grown cells first decreased in the early stage of light period, then gradually increased to a maximum value before the dark period, while for the HL-grown cells, the Φ_PSII_ peaked in the late light period and thereafter decreased towards the end of light period.

### Relationship with daily light doses

**Fig 6:**
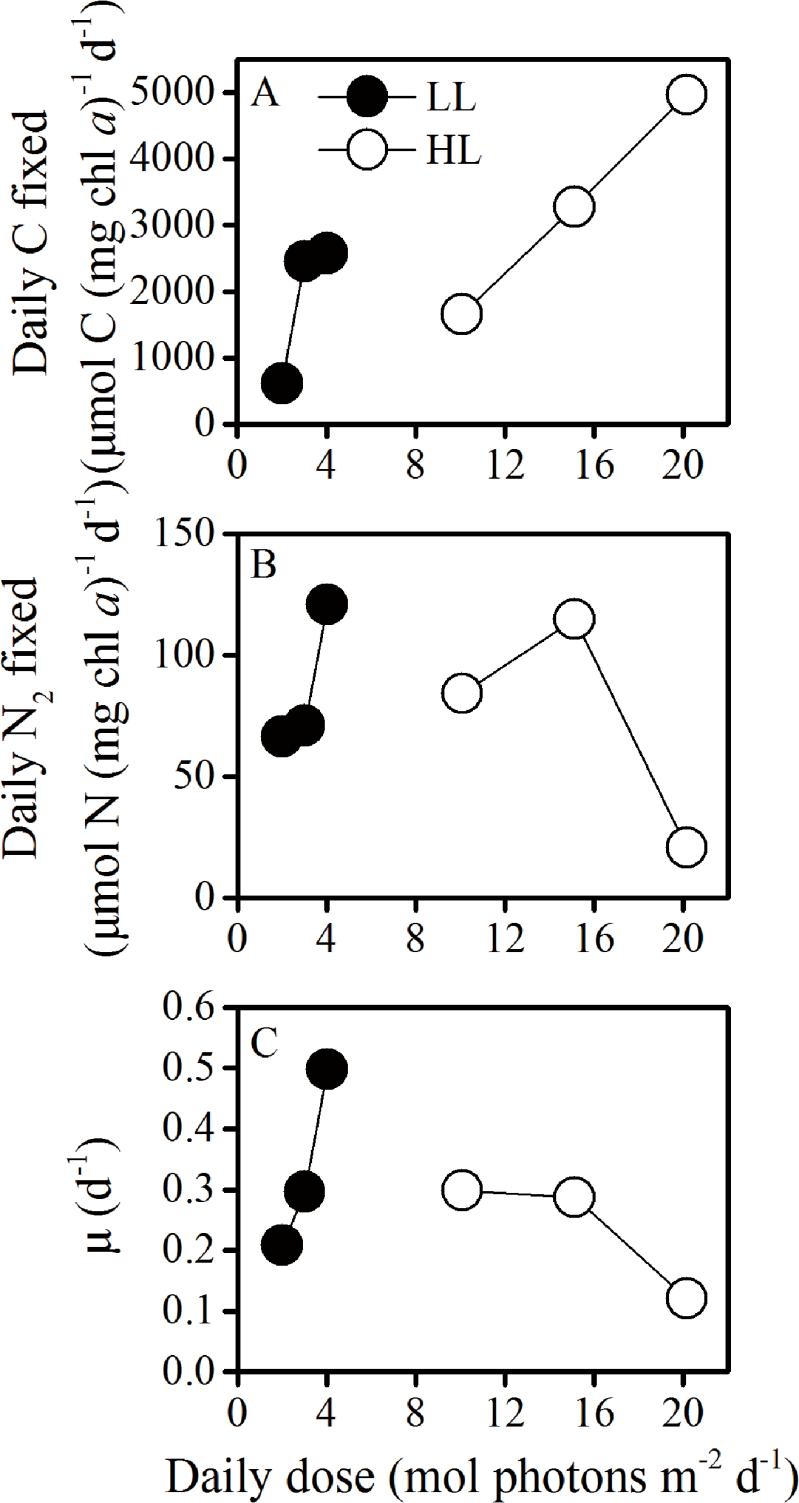
Relationship with daily light doses. Correlations of light doses with (A) daily amount of carbon fixed, (B) daily amount of N_2_ fixed and (C) growth rate in the cells grown under of low (LL, closed circles) and high (HL, open circles) light levels.

The daily fixed amout of N_2_ and carbon were affected differentially by increased daytime light doses, with the former increasing under LL and decreased under HL with the light doses over 16 mol photons m^-2^ d^-1^ and the latter showing linear increase with increased daytime light doses (Fig 6A and 6B). The specific growth rate increased under LL and decreased under HL with increased levels of daytime light doses exposed under the different light regimes, respectively (Fig 6C).

## Discussion

Our results demonstrated that the different day-night cycles as well as different levels of light doses per day affected the diurnal performance of N_2_ fixation and carbon fixation in *Trichodesmium*. Temporal segregation of N_2_ fixation from photosynthetic carbon fixation was evidenced under all day-night regimes, with different magnitudes under different levels or regimes of light. Elongation and shortening of light period affected not only the maximal rates and daily fixed amount of N_2_ and carbon as well as the timing of maximal N_2_ fixation, which occurred at the end of the shortest but at early phase of the longest light period. The imbalance between daily carbon and nitrogen fixation was suggested to cause the changes in the growth rate under different light regimes.

N_2_ fixation in *Trichodesmium* spp. is known to follow a circadian rhythm that developed after illumination, peaked at the middle and decreased near the end of the light period [13, 16]. In this study, N_2_ fixation rates peaked during the light periods at different timings: relatively early in 16L:8D, at midday in 12L:12D, and late in 8L:16D day-night cycles (Fig 2), reflecting a functional plasticity of the N_2_ fixation. Daily *de novo* synthesis, transcriptional and post-translational levels of nitrogenase [13, 24–26] could be expected to display similar plasticity. Buildup of cellular glutamine and glutamate pools [27] must have led to a feedback inhibition to N_2_ fixation through glutamine /glutamate (GS/GOGAT) pathway [28, 29]. On the other hand, the fact that N_2_ fixation peaked at the end of light period and extend to dark period under the 8L:16D regime (Fig 2B) implies an additional N requirement with extra energetic drive at the late light period. Although a rapid decrease in nitrogenase activity after a short dark incubation was observed [16], energy reserve from photosynthesis in the light period was supposed to be capable of supporting N_2_ fixation during early night period [30]. A unicellular N_2_-fixing cyanobacterium *Cyanothece* sp. showed maximal N_2_ fixation at different timings during dark period when transferred to different light-dark regimes [31]. Obviously, both daytime and nighttime N_2_-fixing diazotrophs alter their diel N_2_ fixation performance when grown under different day-night cycles.

Temporal segregation of photosynthestic O_2_ evolution and N_2_ fixation has been reported in *Trichodesmium* [17] and in a few other filamentous [32, 33] and unicellular non-heterocystous cyanbacteria [34]. Lower net O_2_ evolution coincides with lower CO_2_ fixation at mid-day [17]. In this study, such a temporal segregation of N_2_ fixation from photosynthetic carbon fixation was observed under all day-night regimes, but the minimal carbon fixation and maximal N_2_ fixation did not occur at the same time, with the peaks of N_2_ fixation occurring prior to that of carbon fixation (Figs 2,3). Such a time lag between minimal carbon fixation and maximal N_2_ fixation indicates an initial photosynthesis-dependent N_2_ fixation following with a subsequent photosynthetic suppression with peaked N_2_ fixation, which implies N dependency of continuous operation of photosynthetic machinery. This seems to reflect, a time-delay in *nif* gene expression due to photosynthetic metabolic feedback. Since regulation of nitrogenase synthesis by O_2_ occurs at the level of *nif* gene transcription [35], suppression of photosynthesis may open a window for N_2_ fixation in the following hours, while the metabolic feedback to gene expression might be on a slower time scale (hours) [36, 37].

For the photosynthetic performance, a correlation between the carbon fixation and the rETR was observed (Figs 3 and 5), since photosynthetic electron transport or energy transfer is the direct driver for the carbon fixation [38]. Nevertheless, in cyanobacteria, the photosynthetic and dark respiratory electron transport chain share the same plastoquinone pool [39], thus the respiration electron transport during the light period affects the actual redox state of the plastoquinone pool, which in turn affect the quantum yield of PSII (ΦPSII) [19]. Therefore, the fluctuation amplitude of rETR was lower than that of carbon fixation (Figs 3 and 5). In addition, carbon fixation is not the only sink of electrons generated from light reaction. N_2_ fixation, Mehler reaction [9], photorespiration [40] and inorganic carbon uptake can also compete for energy [41]. The ΦPSII as well as rETR showed higher values in short-day regime (8L:16D) of HL, coinciding with higher N_2_ fixation rate, which appeared to support the proposition that the N_2_ fixation may be a major sink of photosynthetic electrons [42]. The phenomenon that rETR values in the 8L:16D regime were higher compared to other L:D regimes indicates a photosynthetic strategy by which relatively more photons could be captured within shorter period, and the mechanisms involved should be studied in future works.

The daily fixed amount of carbon and N_2_ of LL-grown cells both increased with increased daily light doses (Fig 6A and 6B), leading to increased growth rates (Fig 6C). Such a phenomenon indicates light energy limitation in the LL-grown cells. In the HL cultures, the daily amount of carbon fixed increased with increasing light doses, while the daily amount of N_2_ fixed decreased with light dose over 16 mol photons m^-2^ d^-1^, reflecting a severe inhibition of N_2_ fixation of HL-grown cells under prolonged light regime (Fig 2C), which must have resulted in reduced growth rates (Figs 1 and 6C). Previous studies on *Trichodesmium* IMS101 showed that nitrogenase activity saturates at light levels of 200–400 μmol photons m^-2^ s^-1^ in the cells grown at 50 or 500 μmol photons m^-2^ s^-1^ [42], while other strains of this genus saturates at lower light levels [43, 44], with saturation of nitrogenase activity occurring prior to that of photosynthetic electron transport [42]. Since the HL level is highly over the saturating light levels for the growth and nitrogen fixation according to previous studies [19, 42], photosynthetic O_2_ evolution was nearly saturated under this light level [45], therefore, accumulation of photosynthetically evolved O_2_ might have harmed nitrogenase and decreased nitrogen fixation. Additionally, long-day condition can also cause photoinhibition due to increased reaction oxygen species [46]. In the present study, longer exposure to high light induced severe inhibition to N_2_ fixation but not to the photosynthetic carbon fixation (Figs 2 and 3), indicating differential energy demand between C and N_2_ fixations in *Trichodesmium*. Nitrogenase gene expression and N_2_ fixation were shown to severely wither under continuous light [13], and the inhibition of N_2_ fixation under continuous light depends on light intensities [47]. In the present work, both light intensity and doses affected diurnal performance of photosynthesis, growth rates and N_2_ fixation. In view of the relationship of these parameters with light doses, carbon fixation increased with increasing growth light doses, while N_2_ fixation increased under the low to moderate levels but decreased under the high levels of light doses (above 16 mol photons m^-2^ d^-1^) (Fig 6).

Because of the imbalance between carbon and N fixation, the C:N_2_ fixation ratio ranged from 9 to 239 (mol: mol) (Table 1). The lowest C:N_2_ ratio was found in the LL-grown cells under short-day condition, while the highest C:N_2_ fixation ratio was found in the HL-grown cells under long-day condition, which could be due to higher rates of photosynthetic carbon fixation and lower levels of N_2_ fixation over the longer light period (Fig 3C). While C: N_2_ fixation ratios are often higher than the Redfield ratio [48, 49] and can be up to 400~700 in *Trichodesmium* [50, 51], the observed uncoupling of carbon and N_2_ fixation in our study might be due to exudation of newly fixed C especially in long-day treatment of HL cells [51]. Since growth limitation by internal carbon and nitrogen status follows Liebig’s law (growth is not controlled by the total amount of resources available, but by the scarcest resources), the unbalanced C:N_2_ fixation appeared to be responsible for the reduction of growth rates under the elongated light period to 16 h of the high light cultures (Figs 1 and 6).

While *Trichodesmium* has a maximal biomass density at the depth of 20 to 40 m, where the light intensities would be 600 to 900 μmol photons m^-2^ s^-1^ during noon period [2], it usually forms blooms in surface waters of tropical and sub-tropical oceans with the latitudes between 30°N and 30°S, where the shortest and longest day-time are 10 and 14 h, respectively [52]. However, this ecologically important organism may be drifted to higher latitudes via currents and encounter shorter or longer day-time [52]. 2.9~6.9% of daily N_2_ fixed in the night under short-day regimes should be taken into account when estimating N_2_ fixation by *Trichodesmium* spp. in the field. On the contrary, less N_2_ fixation by 82% under the extended daytime to 16 h under HL should also be considered. Furthermore, different weather conditions also determines daily light doses, so that ecological and biogeochemical roles of this cyanobacterium could be influenced differentially under changing environments in different waters.

## Conclusions

The carbon and N_2_ assimilation as well as growth rate in *Trichodesmium* were light dose dependent, with carbon fixation showing positive and N_2_ fixation having negative relationships with increasing light doses over growth-saturating light levels. The shortest light period led to latest peaking timing of maximal N_2_ fixation, while the longest light period led to N_2_ fixation maximum at early light phase, with photosynthetic carbon fixation showing inverse patterns. The N_2_ fixation extended to dark period when the cells were grown under 8L:16D regime. These results indicate that N_2_ fixation was regulated by light energy supplies in addition to circadian rhythms. The imbalance in C:N_2_ assimilation ratio under different levels of light energy supplies correlated with growth rates, with the maximal growth rate at about 20–30 and the minimal growth at over 200 C:N_2_ ratios.
